# An Ethanolic Extract of Ampelopsis Radix Exerts Anti-colorectal Cancer Effects and Potently Inhibits STAT3 Signaling *In Vitro*

**DOI:** 10.3389/fphar.2017.00227

**Published:** 2017-04-28

**Authors:** Tao Su, Jing-Xuan Bai, Ying-Jie Chen, Xin-Ning Wang, Xiu-Qiong Fu, Ting Li, Hui Guo, Pei-Li Zhu, Yue Wang, Zhi-Ling Yu

**Affiliations:** ^1^Consun Chinese Medicines Research Centre for Renal Diseases, School of Chinese Medicine, Hong Kong Baptist UniversityKowloon Tong, China; ^2^Center for Cancer and Inflammation Research, School of Chinese Medicine, Hong Kong Baptist UniversityHong Kong, China; ^3^HKBU Shenzhen Research Institute and Continuing EducationShenzhen, China

**Keywords:** Ampelopsis Radix, colorectal cancer, STAT3, apoptosis, migration, invasion

## Abstract

Colorectal cancer (CRC) is a leading cause of cancer-related morbidity and mortality worldwide. Signal transducer and activator of transcription 3 (STAT3) signaling is constantly activated in CRC, and has been proposed as a pathogenic factor and a therapeutic target of CRC. Ampelopsis Radix (AR), a traditional Chinese medicinal herb, possesses low toxicity and has long been used clinically for the treatment of cancers including CRC. Some constituents of AR have been reported to exert anti-cancer properties by targeting STAT3. However, the anti-CRC mode and mechanism of action of AR have not been fully elucidated. Here, we investigated the involvement of STAT3 signaling in the anti-CRC effects of AR. Results showed that AR reduced cell viability, induced cell apoptosis, and suppressed cell migration and invasion in human HCT-116 and SW480 CRC cells. Mechanistic studies showed that AR potently suppressed STAT3 and Src phosphorylation, and inhibited STAT3 nuclear localization in cultured CRC cells. AR also downregulated the expression of STAT3 target genes Mcl-1, Bcl-xL, and MMP-2 that are involved in cell survival and mobility. Moreover, the cytotoxic effect of AR was diminished by overexpressing STAT3C, a persistent active variant of STAT3. In conclusion, AR exerted anti-CRC effects *in vitro* and these effects are at least in part attributed to the inhibition of STAT3 signaling. Our findings provide a molecular justification for the traditional use of AR in treating CRC, and a pharmacological basis for developing AR-derived modern anti-CRC agent(s).

## Introduction

Colorectal cancer (CRC) is one of the prevalent cancer types worldwide, with an estimated 1.4 million new cases diagnosed each year (Torre et al., 2015). Although a variety of therapies are available for treating CRC, the prognosis for CRC remains poor. The 5-year survival rate of CRC is still less than 65% (Garborg, 2015). Most chemotherapeutics have failed in CRC treatment because of their limitations such as toxicities and low response rate (Rodrigues et al., 2016). Effective and safe targeted chemotherapeutic agents for CRC management are needed. Signal transducer and activator of the transcription 3 (STAT3), whose constitutive activation is frequently detected in various human cancers including CRC, has been proposed as a pathogenic factor of CRC (Ji et al., 2016) and a target for CRC treatment (Ma et al., 2004; Kusaba et al., 2005). Mutations in the pattern recognition receptors like NLRs or AIM2 have been implicated in susceptibility to CRC, and STAT3 activation drives the progression of CRC in susceptible hosts (Karki et al., 2016, 2017; Man et al., 2016). STAT3 silencing in tumor models induces tumor cell death, leading to tumor regression (Lin et al., 2011), and inhibits CRC metastasis (Zhao et al., 2016).

Ampelopsis Radix (AR), a traditional Chinese medicinal herb, is the dried root tuber of *Ampelopsis japonica* (Chinese Pharmacopoeia Commission, 2015). AR was first documented in the earliest currently available book on Chinese medicinal materials Shennong’s herbal classic (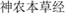) written 2,000 years ago. It has been traditionally used to treat various diseases such as *Yongzhong* (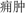), *Luoli* (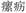), *Changfeng* (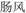), that are nowadays diagnosed as cancers including CRC. In modern clinical Chinese medicine practice, AR is commonly used for treating CRC, and no AR-related toxicity was observed (Zhang et al., 1996; Yang et al., 1999; Huang, 2001; Zhang and Shi, 2005). Pharmacological studies have demonstrated that AR has anti-cancer properties against several cancers including CRC (Lee et al., 1998; Jia and Zhang, 2011; Zhang et al., 2012, 2013; Nho et al., 2015). Some constituents occurring in AR, e.g., gallic acid (Subramanian et al., 2016), catechin (Haratifar et al., 2014), and epicatechin (Kim et al., 2012), have been reported to exert anti-cancer effects by inhibiting STAT3 signaling (Leong et al., 2009; Shay et al., 2015; Phan et al., 2016). However, the anti-CRC mode and mechanism of action of AR are not fully understood. In this study, we investigated the anti-CRC effects of an ethanolic extract of AR and explored the involvement of STAT3 signaling in the effects.

## Materials and Methods

### Chemicals and Regents

3-(4,5-dimethylthiazol-2-yl)-2,5-diphenyltetrazolium bromide (MTT) and DMSO were purchased from Sigma Chemicals Ltd. (St. Louis, MO, USA). Antibodies against GAPDH and SP1 were purchased from Santa Cruz Biotechnology (Santa Cruz, CA, USA), goat anti-rabbit IgG, goat anti-mouse IgG and protein marker were supplied by Bio-Rad (Hercules, CA, USA), and the other antibodies were obtained from Cell Signaling Technology (Beverly, MA, USA). All materials for cell culture were obtained from Life Technologies Inc. (GIBCO, USA).

### Herbal Samples

AR, originated from Jiangsu province, China, was purchased from the Chinese Medicine Clinic of the Hong Kong Baptist University, and authenticated in accordance with the corresponding monograph in the 2015 edition of CP by the corresponding author. Voucher specimen of AR (No. 20151201) was deposited at the School of Chinese Medicine, Hong Kong Baptist University.

Preparation of AR extract: powdered AR was reflux-extracted twice with 30% ethanol (1:10, w/v) for 2 h each. Combined extracts were filtered after cooling and then concentrated under reduced pressure to remove the solvent. The powdered extract (yield: 1.71%) was obtained by lyophilizing of the concentrated samples with a Virtis Freeze Dryer (The Virtis Company, New York, NY, USA). HPLC analysis showed that gallic acid, catechin, and epicatechin were in the extract (we name the extract AR hereafter) (**Supplementary Figure S1**). The contents of these three compounds in AR were 0.23, 1.25, and 0.18 g/kg, respectively.

### Cell Culture

The human CRC HCT-116 and SW480 cells were obtained from the ATCC (Manassas, VA, USA). Cells were cultured in DMEM (GIBCO, USA) supplemented with 5% heat-inactivate USA) and 1% penicillin/streptomycin at 37°C in a humidified 5% CO_2_ atmosphere.

### Cell Viability Assay

The cytotoxic effect of AR on CRC (HCT-116 and SW480) cells and normal human colon mucosal epithelium (NCM460) cells were determined by the MTT assay as described previously (Karki et al., 2013a). HCT-116 and SW480 cells were treated with various concentrations of AR (50–600 μg/ml) or vehicle for 24 or 48 h. NCM406 cells were treated for 24 h. The IC_50_ value of AR against each cell line was calculated using the software Origin 4.0 (Microcal, Northampton, MA, USA).

### Apoptosis Assay

HCT-116 and SW480 cells were seeded on 6-well plates (5 × 10^5^ cells/well) and allowed to adhere overnight. Apoptotic effects of AR (50, 100, and 150 μg/ml) on CRC cells were evaluated by AnnexinV/PI double staining according to the manufacturer’s instructions. Briefly, cells were harvested after 48-h treatment and 1 × 10^5^ cells were then incubated in 100 μL labeling solution (5 μL of AnnexinV-FITC, 5 μL of PI, 10 μL of 10X binding buffer, and 80 μL of H_2_O) in darkness at room temperature for 15 min. After that, 400 μL of 1X binding buffer was added to stop the staining reaction. Flow cytometric analyses were performed on a FACSCalibur^TM^ (BD Biosciences, USA) utilizing 10,000 events.

### Cell Migration Assay

The wound healing assay was used to evaluate the ability of cell migration (Jun et al., 2016). When cells were grown to 80–90% confluence in 6-well plates, wounds were created by scratching the confluent cell monolayer using a plastic pipette tip. Cells were then washed with PBS to remove the detached cells and incubated with AR (12.5, 25 μg/ml) or vehicle in serum-free DMEM medium. Migration of cells into the wound area was photographed at 0, 24, and 48 h time points under a microscope (Leica, Germany).

### Cell Invasion Assay

Cell invasion was determined by using BD BioCoat^TM^Matrigel^TM^ invasion chamber (24-well plate, 8-μm pore size) according to the manufacturer’s instruction (Cao et al., 2014). 0.75 mL of DMEM with 10% FBS was added into the lower chamber. 1.5 × 10^5^ cells in 0.5 mL of DMEM-0.1% BSA with AR (25 μg/ml) or vehicle were placed in the upper chamber. Invaded cells (cells on the lower surface of the membrane) in five microscope areas (200× magnification) were counted and imaged by a microscope (Leica, Germany).

### Gelatin Zymography

Matrix metalloproteinase-2 (MMP-2) activity was measured by gelatin zymography. Cells were treated for 24 h with AR (25, 50, and 100 μg/ml) or vehicle in serum-free DMEM medium, and then the supernatant was collected and measured as described previously (Cao et al., 2014). The gelatinase activity was indicated by the clear bands on blue background, the width of the band is considered to be proportional to the enzyme activity.

### Preparation of Cytoplasmic and Nuclear Fractions

HCT-116 and SW480 cells were seeded as described in Section “Apoptosis Assay.” After incubation for 24 h, AR-treated (50, 100, 150 μg/ml) and vehicle-treated cells were collected. The cytoplasmic and nuclear extracts were prepared as described previously (Cao et al., 2014).

### Western Blot Analysis

HCT-116 and SW480 cells were seeded and treated as described in Section “Apoptosis Assay.” Cell extracts were prepared, electrophoresed under denaturing conditions. The proteins were transferred onto polyvinyldifluoridine membranes. The membranes were washed in TBS containing 0.05% (v/v) Tween-20 and incubated over night at 4°C with corresponding primary antibodies. The membranes were then incubated with secondary antibodies and signals were detected by ECL detection reagents (Amersham Biosciences, USA) (Karki et al., 2013b).

### Plasmid Transient Transfection

The constitutive activated STAT3 expression construct STAT3-C Flag pRc/CMV was obtained from Addgene (USA). Transfection of STAT3 plasmid into HCT-116 or SW480 cells were conducted by using Lipofectamine 2000 (Invitrogen, USA) following manufacturer’s protocol (Fu et al., 2014). Cells were transfected with plasmids for 48 h before functional assays were carried out.

### Statistical Analysis

Results were presented as mean ± SD. Data were analyzed by one-way ANOVA followed by the Dunnett’s multiple comparisons. Statistical analyses were carried out using GraphPad Prism version 5.0 (GraphPad Software, San Diego, CA, USA). *p* < 0.05 was considered statistically significant.

## Results

### AR Reduced Viability and Induced Apoptosis in CRC Cells

**FIGURE 1:**
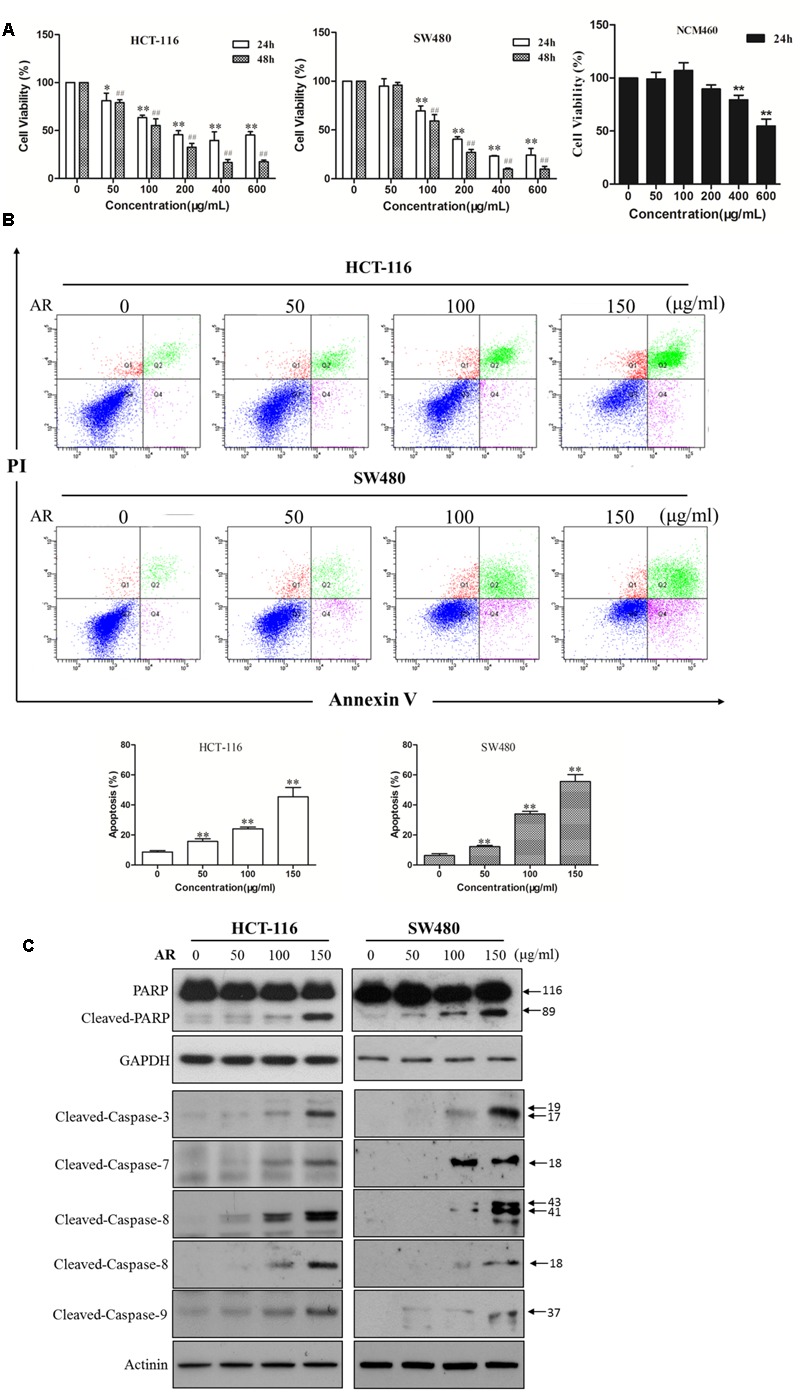
**Ampelopsis Radix decreased viability and induced apoptosis in CRC cells. (A)** Cell viability was measured by the MTT assay. HCT-116 or SW480 cells were treated with various concentrations of AR or vehicle for 24 and 48 h, respectively. NCM406 cells were treated for 24 h. Data were shown as mean ± SD from three independent experiments, ^∗^*p* < 0.05, ^∗∗^*p* < 0.01 *vs*. vehicle (24 h); ^##^*p* < 0.01 *vs*. vehicle (48 h). **(B)** HCT-116 and SW480 cells were treated with various concentrations of AR or vehicle control for 48 h. Apoptosis was analyzed by flow cytometry after Annexin V/PI double staining. The percentage of apoptotic cells was presented as the mean ± SD of three independent experiments, ^∗∗^*p* < 0.01 *vs*. vehicle. **(C)** The expression levels of cleaved-PARP, cleaved-caspase-3, -7, -8, and -9 in HCT-116 and SW480 cells treated with various concentrations of AR or vehicle for 24 h were detected by Western blotting. The representative results were shown.

The cytotoxicity of AR was examined in two human CRC cell lines (HCT-116 and SW480) and a normal human colon mucosal epithelium (NCM460) cell line by using the MTT assay. As shown in **Figure 1A**, AR decreased HCT-116 and SW480 cell viabilities in both time- and dose-dependent manners, with IC_50_ values of 204.0 and 183.9 μg/mL after 24-h treatment, and 123.5 and 128.5 μg/mL after 48-h treatment, respectively. Moreover, the cytotoxicity AR in NCM460 cells was less potent than that in both CRC cell lines. To determine whether AR induced cell apoptosis, the Annexin V/PI double staining assay was used. As shown in **Figure 1B**, AR dose-dependently induced apoptosis in both HCT-116 and SW480 cells. A substantial induction of poly (ADP-ribose) polymerase (PARP), caspase-3, caspase-7, caspase-8, and caspase-9 cleavages in AR-treated HCT-116 and SW480 cells further confirmed the pro-apoptotic effect of AR (**Figure 1C**).

### AR Inhibited CRC Cell Migration and Invasion

**FIGURE 2:**
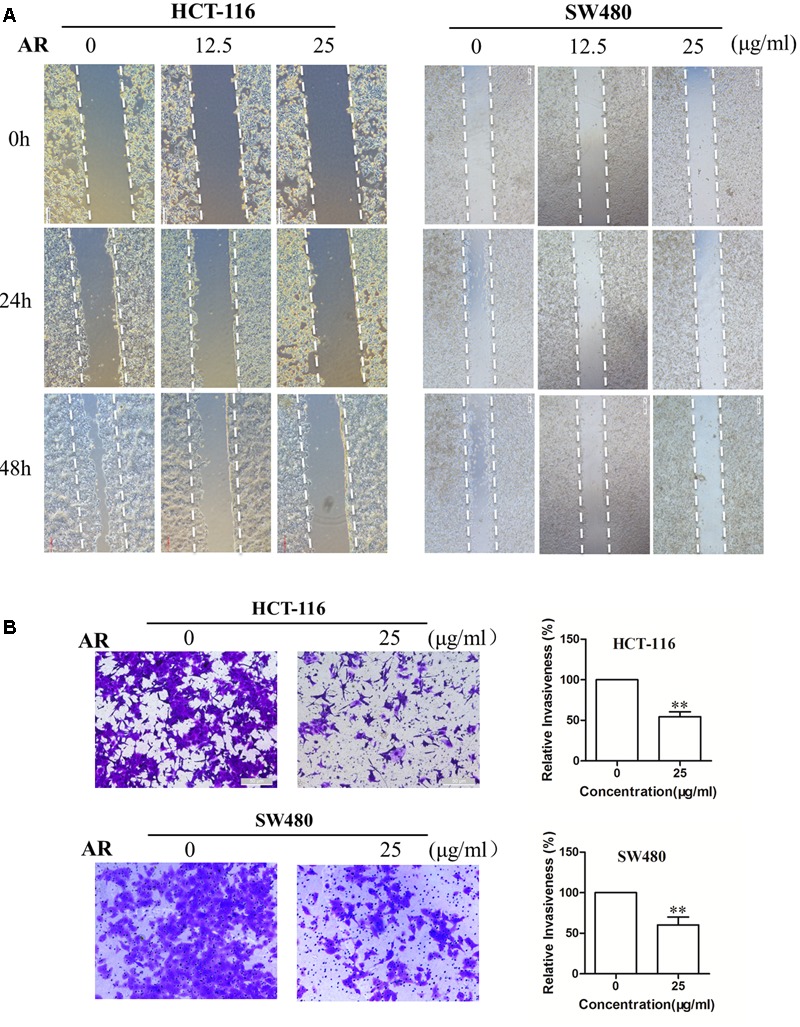
**Ampelopsis Radix impaired the migration and invasion capacities of CRC cells. (A)** HCT-116 and SW480 cells were plated in 6-well plates, and 1 day later, a single scratch was made in the confluent monolayer followed by vehicle or AR treatment, each scratch was photographed after 24- and 48-h treatments. **(B)** HCT-116 cells were allowed to migrate through matrigel coated membrane with 8 μm pores for 48 h in the presence of vehicle or AR. Representative photographs of invaded cells (left) and quantification of invaded cells (right) were shown. Data were presented as mean ± SD from three independent experiments, ^∗∗^*p* < 0.01 *vs*. vehicle.

Wound healing assay and transwell invasion assay were performed to determine the effects of AR on CRC cell migration and invasion, respectively. As shown in **Figure 2A**, after 48 h treatment, AR at the concentration of 25 μg/mL, although did not affect cell viability (**Figure 1A**), significantly inhibited the migratory ability of both HCT-116 and SW480 cells. Likewise, a significant reduction of the cell invasiveness was observed in the AR treatment groups (**Figure 2B**).

### AR Inhibited Src-STAT3 Signaling in CRC Cells

**FIGURE 3:**
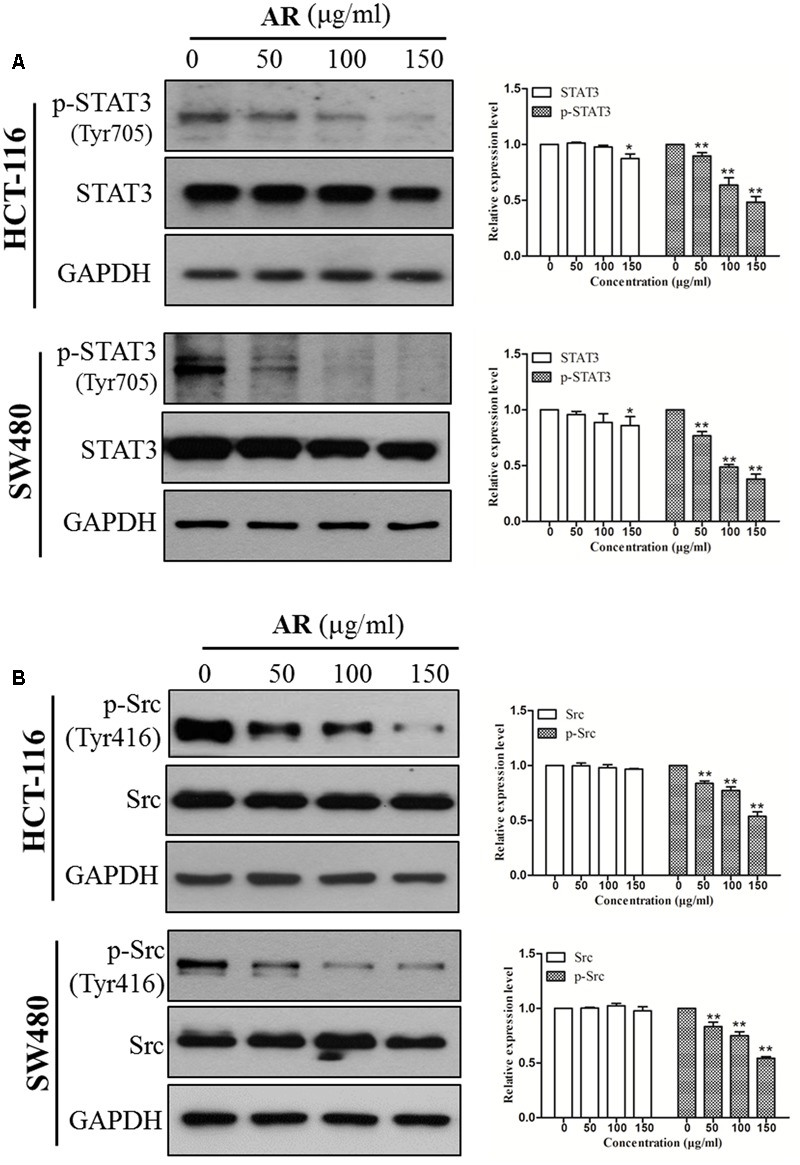
**Ampelopsis Radix suppressed STAT3 phosphorylation in CRC cells.** HCT-116 and SW480 cells were treated with various concentrations of AR or vehicle for 24 h, and then total cell lysates were extracted for Western blot analyses by using antibodies specific to p-STAT3 (Tyr705) and STAT3 **(A)**, p-Src (Tyr416) and Src **(B)**. The representative results (left) and the relative expression levels analyzed by Image J software (right) were shown. Data were presented as mean ± SD from three independent experiments, ^∗^*p* < 0.05, ^∗∗^*p* < 0.01 *vs*. vehicle.

Immunoblotting assay was employed to determine the effects of AR on the phosphorylation/activation of Src and STAT3 in CRC cells. It was found that the levels of phosphorylated STAT3 at the tyrosine 705 (Tyr705) site were reduced in a dose-dependent manner after a 24-h treatment with AR, but the treatment did not affect total STAT3 expression, in both HCT-116 and SW480 cells (**Figure 3A**). Both Src and JAK2 are the upstream tyrosine kinases of STAT3, and they have been found to be constitutively phosphorylated/activated in various cancers including CRC (Kundu et al., 2014). Here, we sought to explore whether AR can inhibit Src and JAK2 activation. Results showed that AR dose-dependently reduced the expression levels of phospho-Src (Tyr416) (**Figure 3B**) but not phospho-JAK2 (Y1007/1008) (data not shown) in both HCT-116 and SW480 cells, suggesting that AR inhibited Src-STAT3 signaling in CRC cells.

### AR Reduced STAT3 Nuclear Localization

**FIGURE 4:**
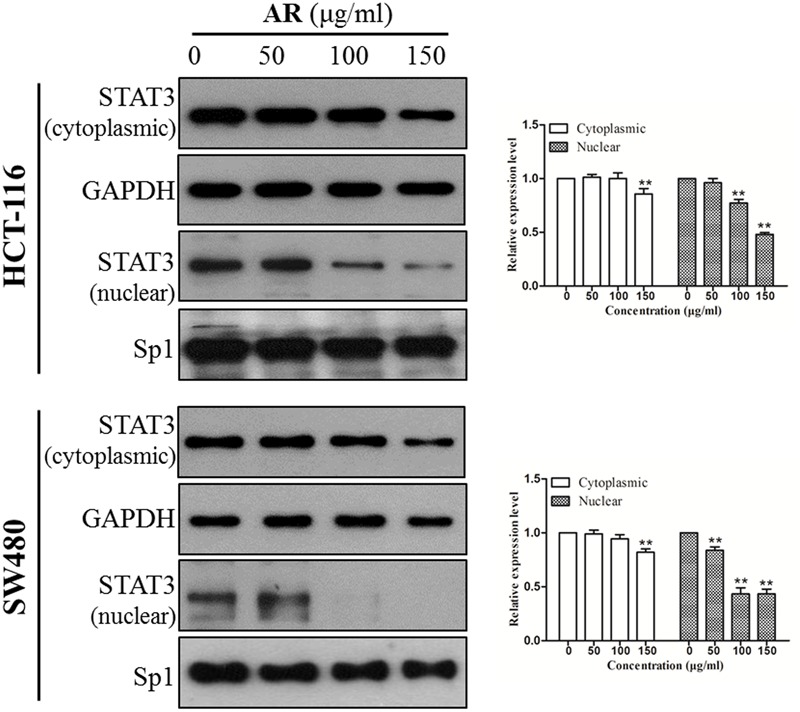
**Ampelopsis Radix decreased STAT3 nuclear localization.** HCT-116 and SW480 cells were treated with indicated concentrations of AR or vehicle for 24 h. Expression levels of STAT3 in cytoplasmic and nuclear extracts were examined by immunoblotting (left) and relative expression levels were analyzed by Image J software (right). GAPDH and SP-1 served as loading controls of cytoplasmic and nuclear extractions, respectively. Data were shown as mean ± SD from three independent experiments, ^∗^*p* < 0.05, ^∗∗^*p* < 0.01 *vs*. vehicle.

STAT3 dimerizes upon tyrosine phosphorylation at site 705, which leads to its nuclear translocation. Inhibition of STAT3 phosphorylation should suppress nuclear localization of STAT3. As expected, the protein levels of STAT3 in nuclear fractions of both HCT-116 and SW480 cells were significantly reduced by AR in a dose-dependent manner (**Figure 4**).

### AR Downregulated STAT3 Target Gene Expression

**FIGURE 5:**
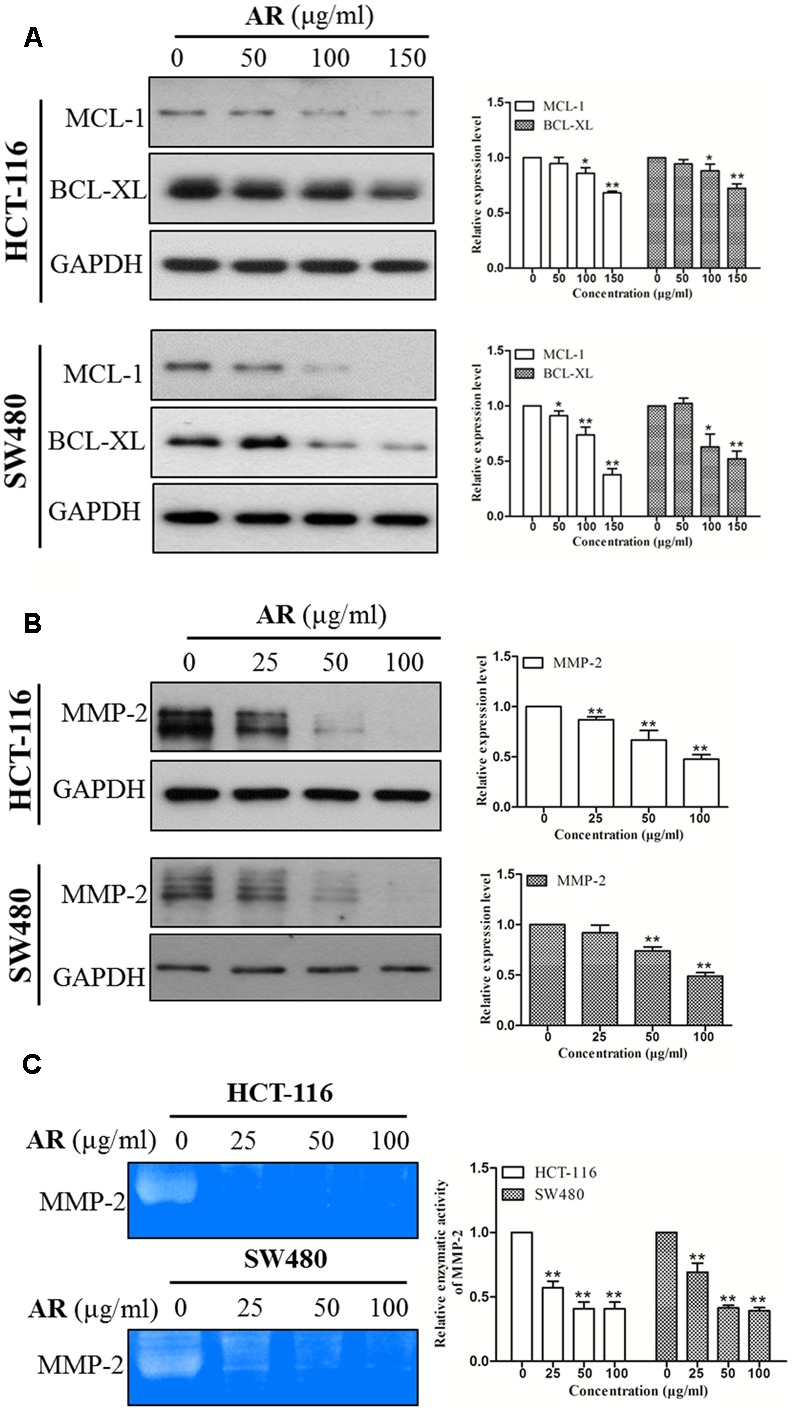
**Ampelopsis Radix downregulated the expression levels of STAT3 target genes.** HCT-116 and SW480 cells were treated with various concentrations of AR or vehicle for 24 h, and then total cell lysates were extracted for Western blot analyses by using antibodies specific to Mcl-1, Bcl-xL **(A)** and MMP-2 **(B)**. Gelatin zymography was used to evaluate the enzymatic activity of MMP-2 **(C)**. The representative results (left) and the relative expression levels analyzed by Image J software (right) were shown. Data were presented as mean ± SD from three independent experiments, ^∗^*p* < 0.05, ^∗∗^*p* < 0.01 *vs*. vehicle.

STAT3 is a transcription factor that directly regulates a panel of tumor-promoting genes (Ao et al., 2015; Lee et al., 2015). We investigate whether AR affected the expression of STAT3 target genes including Mcl-1, Bcl-xL (involve in cell survival), and MMP-2 (involves in cell migration and invasion). As shown in **Figure 5**, AR dose-dependently decreased Mcl-1, Bcl-xL, and MMP-2 protein expression levels, and inhibited MMP-2 enzymatic activity in both HCT-116 and SW480 cell lines.

### Overexpression of STAT3C Diminished the Cytotoxic Effect of AR

**FIGURE 6:**
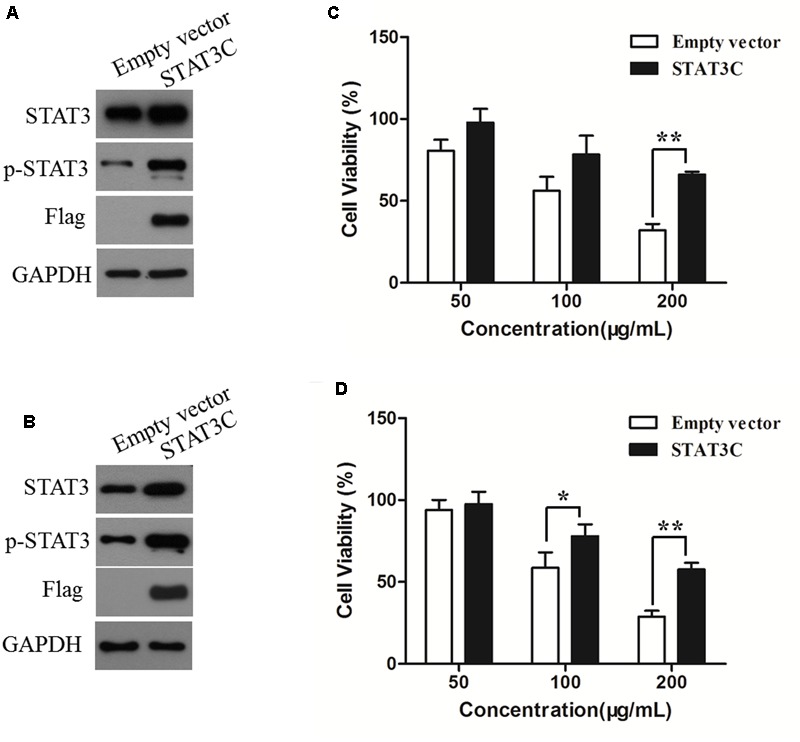
**Overexpression of STAT3C diminished the cytotoxic effect of AR in CRC cells.** HCT-116 **(A)** and SW480 **(B)** cells were transiently transfected with either the STAT3C-expressing construct or the empty vector for 48 h. Protein expression levels were examined by immunoblotting. After plasmid transfection, HCT-116 **(C)** and SW480 **(D)** cells were treated with various concentrations of AR for 48 h, and the cell viability was determined by the MTT assay. Data were the percentages of cell viability of individual AR treatment groups against that of corresponding control groups (in which cells were transfected with the empty vector and STAT3C-harbing plasmid, respectively) but not treated with AR, and were shown as mean ± SD from three independent experiments. ^∗^*p* < 0.05, ^∗∗^*p* < 0.01.

To determine the role of STAT3 inhibition in the anti-CRC effects of AR, HCT-116 and SW480 cells were transiently transfected with an empty vector or a plasmid containing STAT3C, an oncogenic mutant of STAT3 that is constitutively activated without tyrosine phosphorylation (Bromberg et al., 1999). The effects of AR on cell viability of STAT3C- and empty vector-transfected cells were compared. **Figures 6A,B** exemplified a remarkable increase in the expression of total and phosphorylated STAT3 in STAT3C-transfected cells, compared to that in empty vector-transfected cells. The cytotoxic effect of 200 μg/mL of AR was significantly diminished by the ectopic expression of STAT3C in both cell lines (**Figures 6C,D**). In SW480 cells, the cytotoxic effect of 100 μg/mL of AR was also significantly reduced by STAT3C overexpression.

## Discussion

Currently available chemotherapeutics against CRC have various disadvantages such as high price, toxicity, and low efficacy (Braun and Seymour, 2011). These disadvantages highlight the need for exploring safe and effective novel targeted therapeutic agents against CRC. The largely unexplored TCM-based herbs, because of their clinical efficacy and low toxicity, have been recognized as promising candidates for developing alternative/complementary agents to fight malignancies including CRC (Zhang et al., 1996; Yang et al., 1999; Huang, 2001; Zhang and Shi, 2005). AR is one of such herbs. In ancient Chinese medicinal material books, AR was recorded to be non-toxic and able to treat various TCM symptoms that can nowadays be diagnosed as CRC. Current Chinese medicine doctors also prescribe AR, usually in combination with other herbs, to treat CRC. It is noteworthy that no AR-related clinical toxicity was reported. In mice, the oral LD_50_ of AR water extract is as high as 50 g/kg (Lan, 1996). However, no AR-derived anti-CRC modern agent is available. For the purpose of developing AR-derived modern drug(s) to treat CRC, we investigated the anti-CRC effects of AR in cell models. Our data showed that AR was effective. Further studies are warranted to identify bioactive constituents responsible for the anti-CRC activity of AR. We hope to discover compounds with potent anti-CRC efficacy from AR, just like the discovery of artemisinin from a traditional anti-malaria Chinese medicinal herb Herba Artemisiae Annuae, which was inspired from a record in a TCM classic. We also hope to screen out a bioactive components-enriched fraction from the studied ethanol extract of AR for developing a modern anti-CRC agent, because it has been well accepted that natural products with multi-components and multi-targets natures have advantages in managing the complex disease cancer (Hsiao and Liu, 2010).

Previous studies have shown that some compounds occurring in AR exert anti-cancer effects by inhibiting STAT3 signaling (Leong et al., 2009; Shay et al., 2015; Phan et al., 2016). In this study, we found that AR inhibited STAT3 activation/phosphorylation, and the inhibitory effect of AR on CRC cell viability was diminished by ectopic expression of a persistent active variant of STAT3. It is known that, upon phosphorylation, STAT3 dimerizes and translocates into the nucleus to regulate the transcription of its target genes (Johnston and Grandis, 2011). Therefore, we speculated that inhibition of STAT3 phosphorylation might result in reduced STAT3 nuclear localization and decreased expression of genes transcriptionally upregulated by STAT3. Indeed, AR treatment reduced STAT3 nuclear localization and lowered the expression levels of STAT3 targeted Bcl-xL and Mcl-1 that are involved in CRC cell proliferation and survival (Lassmann et al., 2007; Abdulghani et al., 2013), and MMP-2 that is involved in CRC cell mobility (Liu et al., 2015; Ye et al., 2017). Moreover, AR inhibited the phosphorylation/activation of Src, an upstream tyrosine kinase of STAT3. These findings suggest that inhibition of STAT3 signaling contributes to the anti-CRC effects of AR, and support the notion that targeting STAT3 is a strategy for developing novel anti-CRC targeted therapies. One of the major difficulties of developing targeted cancer therapies is to reduce or eliminate the adverse drug reactions resulted from on-target and off-target effects (Kamb et al., 2007; Widakowich et al., 2007) that refer to the effects on normal tissues, and the unexpected toxicities derived from the inhibition of unintended or unknown functions, respectively (Kamb et al., 2007). A number of clinical trials have been approved for using specific STAT3 inhibitors to treat CRC^1^, however, some of them were aborted because of severe adverse effects (Okusaka et al., 2015; Chiba, 2016). AR has long been used in CRC treatment by TCM practitioners in ancient China as well as in modern clinical practice without reported toxicity, and our experimental findings indicate that AR exerted anti-CRC effects *in vitro*, suggesting that the multi-component and multi-target AR may have greater clinical significance compared to specific STAT3 inhibitors.

## Conclusion

In summary, we demonstrated that AR reduced cell viability, induced cell apoptosis and suppressed cell migration and invasion in human CRC cells. Mechanistic studies showed that AR decreased STAT3 and Src phosphorylation, reduced STAT3 nuclear localization, and downregulated the expression STAT3 target genes. Moreover, overactivation of STAT3 decreased the cytotoxic effect of AR in CRC cells. Our results indicate that AR has anti-CRC effects *in vitro*, and these effects are partially due to the inhibition of STAT3 signaling. These findings provide a justification for the clinical application of AR at molecular levels. Together with ancient records, our data also suggest that AR and AR-derived compounds have the potential to be developed as modern alternative and/or complimentary agents for CRC management.

## Author Contributions

TS, J-XB, Y-JC, and X-NW performed the majority experiments. X-QF, TL, HG, and P-LZ participated in several experiments. TS and Z-LY interpreted the data. TS and YW drafted the manuscript. Z-LY supervised the study and finalized the manuscript. All authors have read and approved the final manuscript.

## Conflict of Interest Statement

The authors declare that the research was conducted in the absence of any commercial or financial relationships that could be construed as a potential conflict of interest.

## Footnotes

1 https://clinicaltrials.gov/

## Supplementary Material

FIGURE S1 **High performance liquid chromatography (HPLC) chromatograms of standards and AR. (A)** standards (gallic acid, catechin, and epicatechin); **(B)** AR extract. HPLC analysis was performed to control the quality of AR extract by using an Agilent 1100 series LC system (Agilent Technologies, Santa Clara, CA, USA) coupled with a Grace Alltima C_18_ column (4.6 mm^2^ × 250 mm^2^, 5 μm) maintained at 25°C. Elution was performed with a mobile phase of C (water) and D (acetonitrile). A gradient elution of 4–10% D at 0–10 min and 10–50% D at 10–50 min was employed. The flow rate was 1 mL/min, the injection volume was 10 μL, and the detection wavelength was 215 nm. The HPLC chromatograms showed that gallic acid, catechin, and epicatechin were in AR extract. The contents of gallic acid, catechin, and epicatechin in AR were 0.23, 1.25, and 0.18 g/kg, respectively.

## Abbreviations

AR: Ampelopsis Radix
ATCC: American Type Culture Collection
CP: Chinese pharmacopoeia
CRC: colorectal cancer
DMSO: Dimethyl sulfoxide
DMEM: Dulbecco’s Modified Eagle Medium
FBS: fetal bovine serum
FITC: fluorescein isothiocyanate
HPLC: high performance liquid chromatography
MMP-2: matrix metalloproteinase-2
NLRs: NOD-like receptors
PBS: phosphate buffered saline
PI: Propidium iodide
STAT3: signal transducer and activator of transcription 3
TCM: traditional Chinese medicine
