# Supplementary figures and images for: An Ethanolic Extract of Ampelopsis Radix Exerts Anti-colorectal Cancer Effects and Potently Inhibits STAT3 Signaling In Vitro
Source: Front Pharmacol. 2017 Apr 28;8:227. doi: 10.3389/fphar.2017.00227 (PMC5408070; doi:10.3389/fphar.2017.00227)

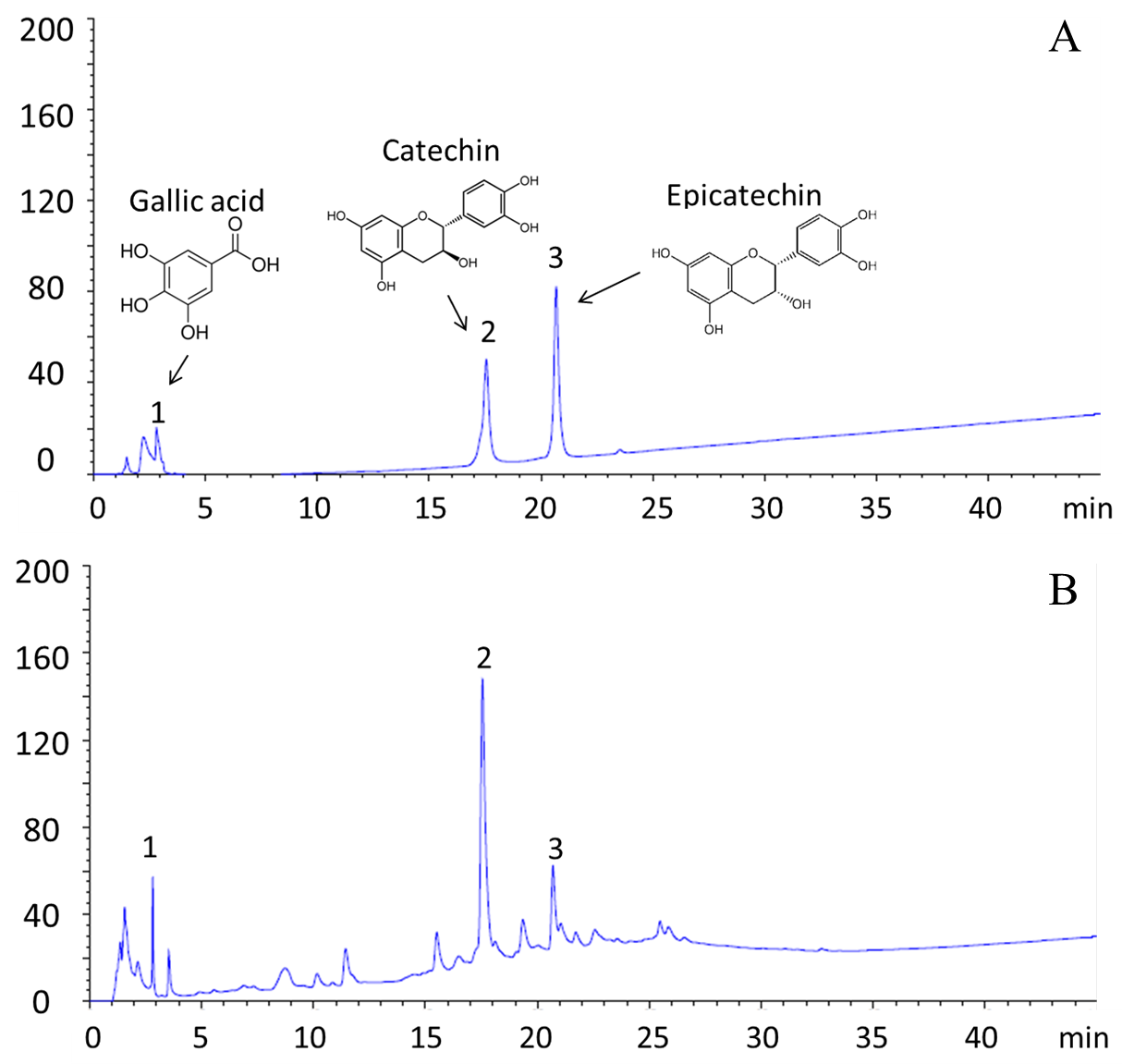

Supplement: FIGURE S1 — High performance liquid chromatography (HPLC) chromatograms of standards and AR. (A) standards (gallic acid, catechin, and epicatechin); (B) AR extract. HPLC analysis was performed to control the quality of AR extract by using an Agilent 1100 series LC system (Agilent Technologies, Santa Clara, CA, USA) coupled with a Grace Alltima C18 column (4.6 mm2 × 250 mm2, 5 μm) maintained at 25°C. Elution was performed with a mobile phase of C (water) and D (acetonitrile). A gradient elution of 4–10% D at 0–10 min and 10–50% D at 10–50 min was employed. The flow rate was 1 mL/min, the injection volume was 10 μL, and the detection wavelength was 215 nm. The HPLC chromatograms showed that gallic acid, catechin, and epicatechin were in AR extract. The contents of gallic acid, catechin, and epicatechin in AR were 0.23, 1.25, and 0.18 g/kg, respectively. [file Image_1.TIF]
